# Do backers prefer crowdfunding or pre-order? An empirical study

**DOI:** 10.3389/fpsyg.2022.984775

**Published:** 2022-11-01

**Authors:** Yuan Zhou, Jie Cui, Nianxin Wang

**Affiliations:** ^1^School of Management, Jiangsu University, Zhenjiang, China; ^2^School of Management Engineering, Huaiyin Institute of Technology, Huaian, China; ^3^School of Economics and Management, Jiangsu University of Science and Technology, Zhenjiang, China

**Keywords:** crowdfunding, pre-order, product stage, backer preference, propensity score matching

## Abstract

To advertise or estimate demand, many pre-order items appear on crowdfunding platforms. Few studies have explored backers’ preferences between crowdfunding projects and pre-order items. To analyze backers’ preferences, 1,800 technology and innovation campaigns were collected from the *Indiegogo* crowdfunding platform. Using the product stage badge, the campaigns in the concept and prototype stages were treated as crowdfunding projects, while those in the production and shipping stages were labeled pre-order items, resulting in 1,305 crowdfunding projects and 495 pre-order items, respectively. Propensity score matching was leveraged to investigate differences in fundraising outcomes between crowdfunding projects and pre-order items. The results indicate that pre-order items have better fundraising outcomes than crowdfunding projects, suggesting that backers are risk-averse on the crowdfunding platform.

## Introduction

Crowdfunding is a new type of internet-based funding model, allowing entrepreneurial individuals and groups of for-profit, cultural, or social projects to request funding from individuals, often in return for future products, equity, or other forms of rewards for the bakers (Mollick, 2014). In recent years, the crowdfunding market has grown rapidly, and its market size has surpassed that of venture capital, becoming one of the mainstream financing methods today (Belleflamme et al., 2015; Capizzi and Carluccio, 2016; Kim et al., 2022). Depending on the relationship between the creator and the backer, crowdfunding can be one of four types: reward-based, donation-based, lending-based, and equity-based, among which reward-based crowdfunding is the determinant funding mode and represents the majority of the crowdfunding market (Bi et al., 2017).

Compared with the booming global crowdfunding market, the success rate of reward-based crowdfunding projects is not yet high. For example, Kickstarter, the world’s largest incentive crowdfunding platform, has launched 555,398 crowdfunding projects, of which only 219,000 have been successfully funded, yielding a success rate of 39.64% (Kickstarter, 2022). Among the 333,435 unsuccessful projects, 56,038 projects raised zero funds, accounting for 16.81%, while 273,299 projects raised less than 20% of their funding goal, accounting for 81.96%. These figures indicate the difficulty of successful crowdfunding fundraising.

Understanding backers’ preferences is a prerequisite for improving the success rate of crowdfunding projects. In fact, two different types of projects are represented on the reward-based crowdfunding platform. One is innovative projects in the early stage of product development, which are posted on the crowdfunding platform to raise necessary funds for implementation. These are typical crowdfunding projects (Guan, 2016), for which the backers are similar to venture capitalists (Mollick and Robb, 2016). The other is projects at the stage of mass production or that are ready to be shipped, which are posted on crowdfunding platforms to target consumers in advance or to promote the products. These are pre-order items (Zheng et al., 2014), and their backers are similar to consumers (Hemer, 2011; Cholakova and Clarysse, 2015; Colombo et al., 2015). Currently, most reward-based crowdfunding platforms allow the release of both types of projects. The most typical is the first Chinese reward crowdfunding platform, Demohour, which supported the release of both crowdfunding and pre-order items at the initial stage of its launch, but suddenly announced that it had transformed into a limited-time pre-order model in August 2014.

Scholars have conducted numerous studies on the emerging funding model of crowdfunding, covering the factors influencing crowdfunding success (Mollick, 2014; Colombo et al., 2015; Yang and Hahn, 2016; Wang et al., 2018), the behavioral patterns of crowdfunding backers (Zhang and Liu, 2012; Burtch et al., 2016; Kuppuswamy and Bayus, 2017a; Wang et al., 2021), and the governance strategies of the crowdfunding platform (Kraut and Resnick, 2011; Gerber et al., 2012; Burtch et al., 2015; Kim et al., 2022). However, there is no literature to investigate backers’ preferences for either crowdfunding projects or pre-order items. Preferences indicate each backer’s appropriate expectation for a specific project in the context of crowdfunding (Du et al., 2021). More specifically, when the crowdfunding project meets a backer’s preferences, the backer is more likely to be persuaded (Wang et al., 2021). By contrast, when the pre-order item fails to meet a backer’s preferences, it might decrease the persuasiveness of the campaign. Therefore, it is important to identify the preferences for either crowdfunding projects or pre-order items in predicting crowdfunding performance.

To examine backers’ preferences for crowdfunding projects or pre-order items, we collected 1,800 projects from Indiegogo, one of the largest crowdfunding platforms in the world. Using the product stage badge, the campaigns in the concept and prototype stages were treated as crowdfunding projects, while those in the production and shipping stages were labeled pre-order items, resulting in 1,305 crowdfunding projects and 495 pre-order items, respectively. Propensity score matching (PSM) was leveraged to investigate the differences in fundraising outcomes between crowdfunding projects and pre-order items. The results indicate that pre-order items produce better fundraising outcomes than do crowdfunding projects, suggesting that backers are risk-averse on the crowdfunding platform.

The study makes two major contributions to the crowdfunding literature. First, it extends the determinants of crowdfunding performance from project and creator characteristics to backer preferences by investigating the performance differences between pre-order items and crowdfunding projects. Second, this research contributes to the effectiveness of the governance mechanism of crowdfunding platforms by empirically examining the effects of the product stage badge on fundraising outcomes. These two theoretical contributions are grounded in the empirical analysis of a special dataset that has not been traced in the crowdfunding literature.

The reminder of this paper proceeds as follows. We next present differences between pre-order and crowdfunding, and develop the hypothesis. Then, we describe the research methodology and report the findings. Finally, we discuss the findings, the theoretical and practical implications, and the limitations.

## Crowdfunding vs. pre-order

### Crowdfunding

Depending on the relationships between the creator and the backers, crowdfunding is divided into four types, namely, reward-based, donation-based, lending-based, and equity-based (Mollick, 2014). Among them, lending-based and equity-based crowdfunding are traditional investment mechanisms, and backers often expect financial returns. Regarding lending-based crowdfunding, creators and backers are equivalent to a lender and borrower; meanwhile, equity-based crowdfunding is similar to traditional venture capital in that it is an entrepreneur–investor relationship. For donation-based crowdfunding, backers do not expect anything in return and play the role of a philanthropist for creators. Reward-based crowdfunding dominates online crowdfunding; here, the individual or company initiating the project is termed the “creator” and the backer usually plays the role of an early customer or partner, rather than simply that of an investor. Reward-based crowdfunding was chosen for this study because it is currently the most dominant mode of crowdfunding and has been widely used for empirical analysis regarding the themes described immediately below.

First, crowdfunding performance is determined by a variety of factors. In a crowdfunding market with information asymmetry, backers tend to combine multiple information to judge the quality of projects to make investment decisions (Burtch et al., 2013; Courtney et al., 2017; Wang et al., 2021). Studies have shown that crowdfunding performance is influenced by many factors, including (1) project description information (Mollick, 2014; Beier and Wagner, 2015; Colombo et al., 2015), such as funding target, funding duration, length of project description, spelling errors, inclusion of videos, and so on; (2) creator experience (Wessel et al., 2015; Yang and Hahn, 2016), such as experience as creator and experience as backer; and (3) interaction between creators and backers (Mollick, 2014; Agrawal et al., 2015; Wang et al., 2018, 2021), such as project updates, backer comments, and creator replies.

Second, previous funding information influences the funding behavior of later backers in the crowdfunding ecosystem. Based on the behavioral characteristics exhibited by the backers, the existing literature summarizes the backer behaviors, including (1) herding behavior (Zhang and Liu, 2012; Agrawal et al., 2014; Wang et al., 2021), which refers to the number of existing backers and the amount of investment positively influencing the motivation of potential backers; (2) diffusion of responsibility behavior (Hardy and Van, 2006; Burtch et al., 2013), whereby the number of existing backers and the amount of investment negatively affects the motivation of potential backers; (3) local preference behavior (Agrawal et al., 2015; Lin and Viswanathan, 2016), which refers to the preference of backers to choose projects that are close to offline funding; and (4) information hiding behavior (Burtch et al., 2016; Kuppuswamy and Bayus, 2017b), which refers to backers’ choice to hide their identities and funding information when funding.

Third, refining and redesigning crowdfunding platforms to improve the user experience can attract more users and maintain the engagement of the platform community. The literature has examined the influence of platforms’ threshold mechanisms (raising funds to reach a goal before receiving the funds raised) (Burtch et al., 2018), privacy protection settings (option to hide identity and funder information) (Burtch et al., 2015), risk information disclosure (Kim et al., 2022; Liu et al., 2022), the level settings in the rewards program (Xiao et al., 2014), and crowdfunding community building (Hui et al., 2014; Saura et al., 2022a). The results show that continuous improvement in the design of various mechanisms of crowdfunding platforms has contributed to the development of the platforms.

### Pre-order

Pre-order is the sale model in which the seller accepts orders from customers and guarantees the availability of the product before the official launch of the product or service (Fay and Xie, 2010; Saura et al., 2021, 2022b). In recent years, with the rise of major e-commerce platforms, people’s consumption views and patterns have also changed, and online pre-orders have begun to present an advantage (Mukherjee et al., 2021; Huang et al., 2022). Pre-order can be divided into three types, namely, origination, discount, and scarcity. Origination pre-order means the pre-order products are newly developed, with a certain degree of innovation, and have not officially entered the market. Consumers who participate in such products are usually technology enthusiasts or loyal fans of a brand. Discount pre-order is mainly found in the shopping festivals of major domestic e-commerce platforms, where pre-order products are sold at a lower price than the normal sale or with free gifts to attract consumers to buy at a certain time. Scarcity pre-order is used in industries where supply is certain and demand is variable. In most cases, demand exceeds supply, and consumers need to purchase goods or services in advance, such as concert tickets, high-speed rail tickets, and movie tickets. The existing research studies on pre-order mainly focus on the following three areas.

First, the pre-order model improves the accuracy of consumer demand forecasting. One important reason for the development of pre-order is the uncertainty of consumer demand and the difficulty for companies to obtain information about consumers’ real demands (Wei and Zhang, 2018). Weng and Parlar (1999) were the first to propose that sellers could entice customers to buy in advance by selling at a discount, thereby forecasting demand based on pre-order data. Moe and Fader (2003) built a model to predict product sales based on pre-order data and compared it with other benchmark models, finding that pre-order data performed better.

Second, the pricing strategy in pre-order has been a key issue studied by scholars. In early studies on pre-order, it was believed that consumers are attracted to buy in advance through discount pre-order, and the price of the product in the pre-order stage should be lower than the price at normal sales (Gale and Holmes, 1992). With the development of online pre-order and an increase in consumer empowerment, some scholars have found that premium pre-order appears to be more advantageous under certain circumstances. Wang and Zeng (2016) found that retailers with pre-order strategies outperformed those without pre-order, but under different circumstances, the optimal pricing strategy was different, highlighting that capacity constraints affected the choice of optimal pricing strategy.

Third, consumer behavior in the pre-order model is influenced by a variety of factors. The development of technology has not only driven the advancement of pre-order but also trained consumers to think about their purchases in a way that maximizes the benefits for themselves by considering various factors. This type of consumer is known as the strategic consumer. Li and Zhang (2013) pointed out that consumers’ willingness to pay in advance depends on their evaluation of the product, expectation of future prices, and the availability of the product. Swinney (2011) specifically discussed the strategic buying behavior of consumers, that is, whether consumers preferred the pre-order stage, where the initial value was uncertain, or the formal sale stage, where the value of the product was determined.

### Differences between crowdfunding and pre-order

The literature has mostly studied crowdfunding and pre-order in two different directions, with some scholars arguing that crowdfunding includes features of the pre-order model and referring to this as pre-order crowdfunding (Bi et al., 2017). In fact, although crowdfunding contains the features of pre-order, there are still significant differences between the two different models. As shown in Table 1, this paper compares crowdfunding and pre-order from six perspectives.

**Table 1:** Crowdfunding vs. pre-order.

Differences	Crowdfunding	Pre-order
Relationships	Creators and backers	Retailers and consumers
Returns	Products or other rewards after the successful implementation of the project, while gaining a sense of participation and pride, and the joy of helping others	Obtain goods at discounts, tastings
Arrival	Delayed, or even unfulfilled	Within the appointed time
Motivation	Product consumption, altruism, and sense of social belonging	Preferential policies, early access to product services
Payments	The amount of payment is determined by the backer	Deposit in advance (full or partial payment)
Risks	Fraud, lack of after-sales service, delayed delivery	Price reduction, lower than expected value

First, the relationship established between the participating parties is different. Owing to the lack of laws and regulations governing crowdfunding, project creators may suffer from fraud and malfeasance, which may be manifested in the form of the creator fleeing with the money after successful fundraising or failing to deliver the rewards at the agreed time (Zhao et al., 2019). Conversely, pre-order is based on traditional e-commerce, where a good trust mechanism is built between buyers and sellers, reducing the perceived uncertainty and risks associated with anonymous online exchanges, such as purchasing goods and exchanging personal information (Ba and Pavlou, 2002).

Second, crowdfunding backers are different from pre-order purchasers. After the successful implementation of a crowdfunding project, the backer may receive a project product, a thank you card, or a special acknowledgment, depending on the amount of money contributed. What is more, the backer will feel a sense of participation and pride in the success of the project, as well as the pleasure of helping others (Hemer, 2011; Zheng et al., 2014; Bi et al., 2017). Meanwhile, the pre-order purchaser will receive the product purchased and the satisfaction of enjoying the product discount or the superiority of the product value before others (Bi et al., 2017; Byun et al., 2017).

Third, the waiting time for the two models to receive the physical goods is different. Crowdfunding backers will wait a long time to receive their rewards, and mostly this period will exceed the previously agreed time, or the promised rewards might not even be received. However, in the pre-order model, purchasers feel more secure and usually receive the goods within the agreed time frame (Gerber and Hui, 2013; Bi et al., 2017).

Fourth, participants have different funding or purchasing motives. The ultimate goal of pre-order purchasers must be to obtain the goods, but pre-order represents a better deal for them than a spot sale (Shugan and Xie, 2004). Conversely, crowdfunding backers might be motivated by the desire to help others and achieve a sense of self-worth in addition to obtaining the final product (Gerber and Hui, 2013; Belleflamme et al., 2014; Bretschneider and Leimeister, 2017; Zhang and Chen, 2019).

Fifth, the payment rules are different between the two. Crowdfunding backers decide the amount of investment according to their own wishes and may contribute more than once. Investments are temporarily kept on the platform side and transferred from the platform side to the creator or returned to the backer, depending on the final status of the project (Mollick, 2012). Pre-order purchasers need to pay a deposit in advance, which would be all or part of the selling price of the goods, and the seller then decides when to pay the final payment (Prasad et al., 2011).

Sixth, crowdfunding is riskier. There may be fraudulent projects in crowdfunding, in which the creator raises the money but does not use it for the development of the project. Instead, the creator flees with the money, or does not supply the products to the backer as described, or does not supply a guaranteed after-sales channel. In addition, most crowdfunding projects will be shipped with long delays, meaning that backers receive less value (Zhao et al., 2019). Two main risks exist for consumers in the pre-order model: one, there is no price guarantee for pre-order products and the price may drop when they are officially released; two, the pre-order product has not been released yet and there is no corresponding product on the market to compare it with, so consumers have unrealistically high expectations of them (Nasiry and Popescu, 2012).

The above analysis demonstrates that the crowdfunding model has different characteristics from the pre-order model in six ways, which are relationship, returns, arrival, motivation, payment, and risk. Compared with pre-order, crowdfunding participants’ contributions not only achieve for them the desired items but also lead them to experience feelings beyond the purchase in the process, such as participation, pride, and the joy of helping others. However, crowdfunding is riskier. Unlike pre-order, in which buyers and sellers are involved, there is no good trust mechanism established between the creator and the backer, and owing to the lack of protection, the backer often experiences long product arrival times and even deceptive practices that do not deliver the return goods. When faced with a choice between the two types of projects on the same platform (crowdfunding and pre-order items), as a rational backer, they tend to support the less risky pre-order item for the purpose of risk avoidance (Courtney et al., 2017; Wang et al., 2021; Kim et al., 2022), which leads to better performance of the pre-order item than the crowdfunding project. Therefore, the following hypothesis is proposed in this paper:

***Hypothesis**: Pre-order items perform better than crowdfunding projects on the same platform.*

## Methodology

### Data collection

**Figure 1:**
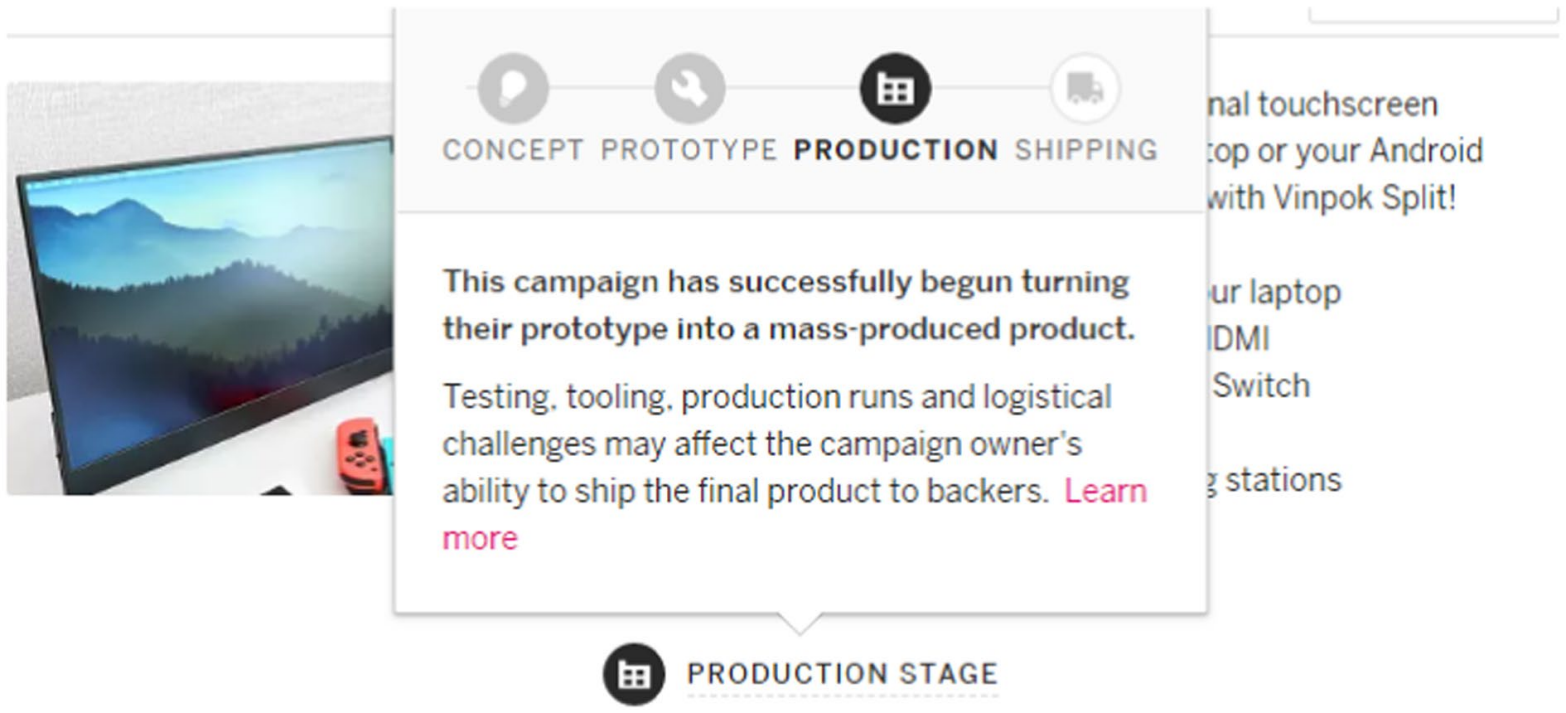
Product stage badge in Indiegogo.

The focus of this paper is on whether crowdfunding backers prefer crowdfunding or pre-order. Our empirical setting is one of the first and largest reward-based crowdfunding platforms worldwide, *Indiegogo*. Indiegogo organizes campaigns into three broad categories: Tech & Innovation, Creative Works, and Community Projects. To help backers understand the development cycle before they contribute, Indiegogo has designed a product stage badge for physical products in the Tech & Innovation category, as illustrated in Figure 1.

The results of the presentation of the project stage badge on the Indiegogo platform are determined by the platform administrators. First, creators from around the world submit their project details to the platform, which reviews the information submitted by the creator (the same information that the backer will later see on the platform). If there is sufficient evidence to prove the current stage of the project, it will be approved; otherwise, it will not be approved, and the creator will be informed of the review result. The final review result will be displayed on the stage tab of the project home page, and the platform allows projects of any stage to participate in crowdfunding. The platform reviews the project stage information, which is more realistic and credible than other available information (project description information, interaction information between the creator and the backer, and creator characteristics) and has greater reference value for backers’ decisions. The project stages present the projects under the Tech and Innovation category with physical products in different stage of product development including concept, prototype, production, and shipping. Descriptions of the different stages are as follows:

#### Concept

Projects in the “Concept” stage have an idea for a physical product that they are planning to create. The creator has only a preliminary idea they will produce and may already have a model (actual function to be realized) or 3D rendering showing the appearance of the product and the function to be realized at this stage.

#### Prototype

The project in the “Prototype” stage has produced a physical object that matches the description of the creator and can successfully demonstrate the main features and functions of the final product.

#### Production

The project in the “Production” stage produces a physical product that matches the creator’s description and is already in mass production ready to be offered to the backer, which can be supported by pictures or even videos of the current stage of the project (e.g., production tools, molds, manufacturing parts, or finished products in a factory).

#### Shipping

Projects in the “Shipping” stage have already produced their final product and have begun shipping it to backers.

It is observed that the concept and the prototype stage are both early stages of product development and therefore have a higher risk, while the production and the distribution stage are later stages of product development and carry a relatively lower risk for being put into the market. Therefore, this paper defines the first two stages as the “crowdfunding stage” and the second two stages as the “pre-order stage.”

The data used for this study were crawled through python to obtain basic information about crowdfunding projects in the Tech & Innovation category on the Indiegogo platform. We found a total of 8,205 projects were launched and closed from January 2015 to November 2018, including a total of 1,836 projects with the project stage tag, while removing projects with a target of less than $500 (Wang et al., 2021). The total valid data analyzed were 1,800 crowdfunding projects.

### Variables

To understand the preferences of backers for crowdfunding projects and pre-order items, this study used the project type (crowdfunding project and pre-order item) as the treatment variable and divided the data into two groups for comparison. Crowdfunding projects in the first two stages were assigned a value of 0; pre-order items in the second two stages were assigned a value of 1. The four outcome variables were divided into four: success rate, funding ratio, number of backers, and average investment, which provided a comprehensive picture of the final performance of the project. The matching variables selected the target amount of funding set by the creator when launching the crowdfunding project, the number of funding days, the number of external websites links, the number of videos and pictures displayed, and the total number of projects launched and supported representing the experience of the creator’s participation. Specific definitions and explanations of the variables are shown in Table 2.

**Table 2:** Measures of variables.

Variables	Measures
**Treatment variable**	
stage12_34	Crowdfunding project is 0, while pro-order item is 1.
**Performance variables**	
Success	The dummy variable takes the value of one if the project has been successfully funded and zero otherwise.
Fund Ratio (RA)	The ratio of total pledge to the target funding goal
Backers (BA)	The number of backers who supported the focal project
Pledge (PL)	Average pledge of one project obtained from each backer
**Matching variables**	
Goal (GO)	The target funding goal specified by the creator
Duration (DU)	The number of days the project campaign stays active
External_URL (URL)	The number of external websites, e.g., E-mail, Facebook, and LinkedIn
Video (VI)	Whether the project description contains video, where 1 = “yes” and 0 = “no”
Pictures (PIC)	The number of pictures included in the project description
Experience as creator (EC)	The number of projects the creator has initiated.
Experience as backer (EB)	The number of projects the creator has supported.

### Descriptive statistics

The data used for analysis in this paper were obtained from the information of Tech & Innovation projects on the Indiegogo crowdfunding platform, containing 1,800 projects with stage labels, including 1,305 projects in the crowdfunding feature stage and 495 projects in the pre-order feature stage. Table 3 provides the descriptive statistics of crowdfunding in different characteristic stages, where it is evident that the mean values of the outcome variables in the experimental group are significantly larger than those in the control group, without considering other factors.

**Table 3:** Descriptive statistics of crowdfunding projects and pre-order items.

Variables	Crowdfunding (*N* = 1,305, stage12_34 = 0)				Pre-order (*N* = 495, stage12_34 = 1)			
	Mean	SD	Min	Max	Mean	SD	Min	Max
Success	0.464	0.499	0	1	0.814	0.389	0	1
RA	3.053	9.023	0	200.514	10.962	30.102	0	523.766
BA	457.651	1688.065	0	41,401	1556.992	3872.056	0	58,665
PL	202.435	483.799	0	10,424	298.416	1219.18	0	26,250
GO	59355.05	322,000	500	9,000,000	31845.76	39355.64	500	502,000
DU	45.969	14.824	1	90	47.166	14.154	1	73
URL	1.343	0.693	0	3	1.325	0.695	0	3
VI	0.884	0.321	0	1	0.98	0.141	0	1
PIC	5.148	6.331	1	99	7.699	8.263	1	89
EC	1.437	1.452	0	39	1.624	1.204	0	10
EB	1.131	4.687	0	85	1.562	4.985	0	55

## Results

### Propensity score matching

Propensity score matching is widely used in analyzing observational datasets to reduce the impact of confounding due to observed covariates (Austin, 2008; Magura et al., 2013; DeFond et al., 2017; Wang et al., 2018, 2021). This technique enabled us to investigate heterogeneous treatment effects in nonexperimental data, based on observed variables. The objective of propensity score matching is to assess the effect of a treatment by comparing observable outcomes (in our case, crowdfunding performance) among treated observations (pre-order items) to untreated observations (crowdfunding projects) matched according to the propensity of being treated.

This study was designed to explore the specific differences between projects in the “crowdfunding stage” and the “pre-order stage,” while avoiding sample selection biases that might have resulted in endogeneity problems, such as mutual causality and neglecting important omitted variables that lead to correlation of explanatory variables with error terms. This paper shows that different categories of crowdfunding projects can have different appeal to backers. Therefore, to address the heterogeneity of individual characteristics, the method of PSM was employed, with crowdfunding stage projects as the control group and pre-order stage projects as the experimental group. Based on the calculated propensity scores, the same or similar individuals were matched from the control group to the experimental group to obtain the effect of being in the crowdfunding stage or the pre-order stage on crowdfunding performance. This study followed the implementation steps suggested by Caliendo and Kopeinig (2008), using the Stata-based PSM method to analyze the collected data.

#### Step 1: Estimating the propensity score

Statisticians Rosenbaum and Rubin (1983) proposed using “propensity scores” to measure distance in 1983, and the method of matching propensity scores as a function of distance was named “propensity score matching.” In their subsequent research, the authors suggested using a flexible logit model to estimate propensity scores (Rosenbaum and Rubin, 1985). In this study, a logit model was used to estimate the predicted probability of each sample item at the pre-order stage, as follows:


Logit(treatij=1)=α+βXij+εij

where *treat_ij_* is a dummy variable indicating the type of project, with a value of 1 assigned to a project being in the pre-order phase and 0 assigned to the crowdfunding phase, and *X_ij_* indicates the matching variable provided in Table 2. Leuven and Sianesi (2003) emphasized that these covariates must be determined prior to the implementation of a project or policy or be exogenously independent of whether or not they are implemented, and that none of these variables are affected by whether or not they are implemented.

#### Step 2: Choosing matching algorithm

In the case of non-exact matching, the matching estimates are generally biased. If one-to-one matching is used, the bias is smaller, but the variance is larger; if the one-to-many matching method is chosen, the variance is effectively reduced because more information is used, but also the bias increases because more distant information is used. Owing to insufficient sample size of the control group, this study chose the K-nearest neighbor matching method of one-to-one matching to correlate the crowdfunding items with the pre-order items.

#### Step 3: Testing overlap and common support

**Figure 2:**
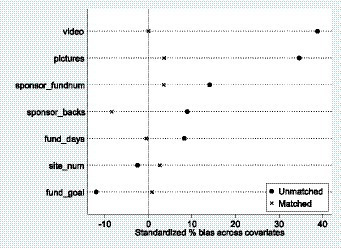
The standardized deviation diagram for each variable (unmatched vs. matched).

To ensure reliability of the PSM effect in the project phase, a balance test was first conducted on the project data. As yet, there is no unified standard deviation threshold for the validity of PSM estimation by scholars. In general, if the standard deviation is smaller, the better the matching effect, and if the absolute value of standard deviation is less than 20%, the PSM is considered more reliable and, vice versa, the matching effect is considered bad (Rosenbaum and Rubin, 1985). The results of the equilibrium test for the item data provided in Table 4 show that the standard deviations of the variables after matching were basically within 10%, which indicates that both groups of item data reached equilibrium. The changes before and after matching of the standardized deviations of the variables, shown in Figure 2, indicate more clearly that the matching effect was good.

**Table 4:** Balance test results of crowdfunding projects and pre-order items.

Variables	Matching	Means		Difference (%)	Difference ratio (%)	*T*-test	
		Treated	Controlled			*T*-value	*p* > |t|
GO	Before matching	31,846	59,355	−12.0	93.6	−1.90	0.058
	After matching	31,846	30,085	0.8		0.66	0.511
DU	Before matching	47.166	45.969	8.3	94.3	1.55	0.122
	After matching	47.166	47.234	−0.5		−0.08	0.939
URL	Before matching	1.3253	1.3425	−2.5	−5.2	−0.47	0.637
	After matching	1.3253	1.3071	2.6		0.42	0.677
VI	Before matching	0.9798	0.88352	38.8	100.0	6.44	0.000
	After matching	0.9798	0.9798	0.0		−0.00	1.000
PIC	Before matching	7.699	5.1479	34.7	89.7	6.99	0.000
	After matching	7.699	7.4364	3.6		0.52	0.601
EC	Before matching	1.6242	1.4368	14.1	75.2	2.56	0.011
	After matching	1.6242	1.5778	3.5		0.42	0.673
EB	Before matching	1.5616	1.131	8.9	5.2	1.71	0.087
	After matching	1.5616	1.9697	−8.4		−1.04	0.299

**Figure 3:**
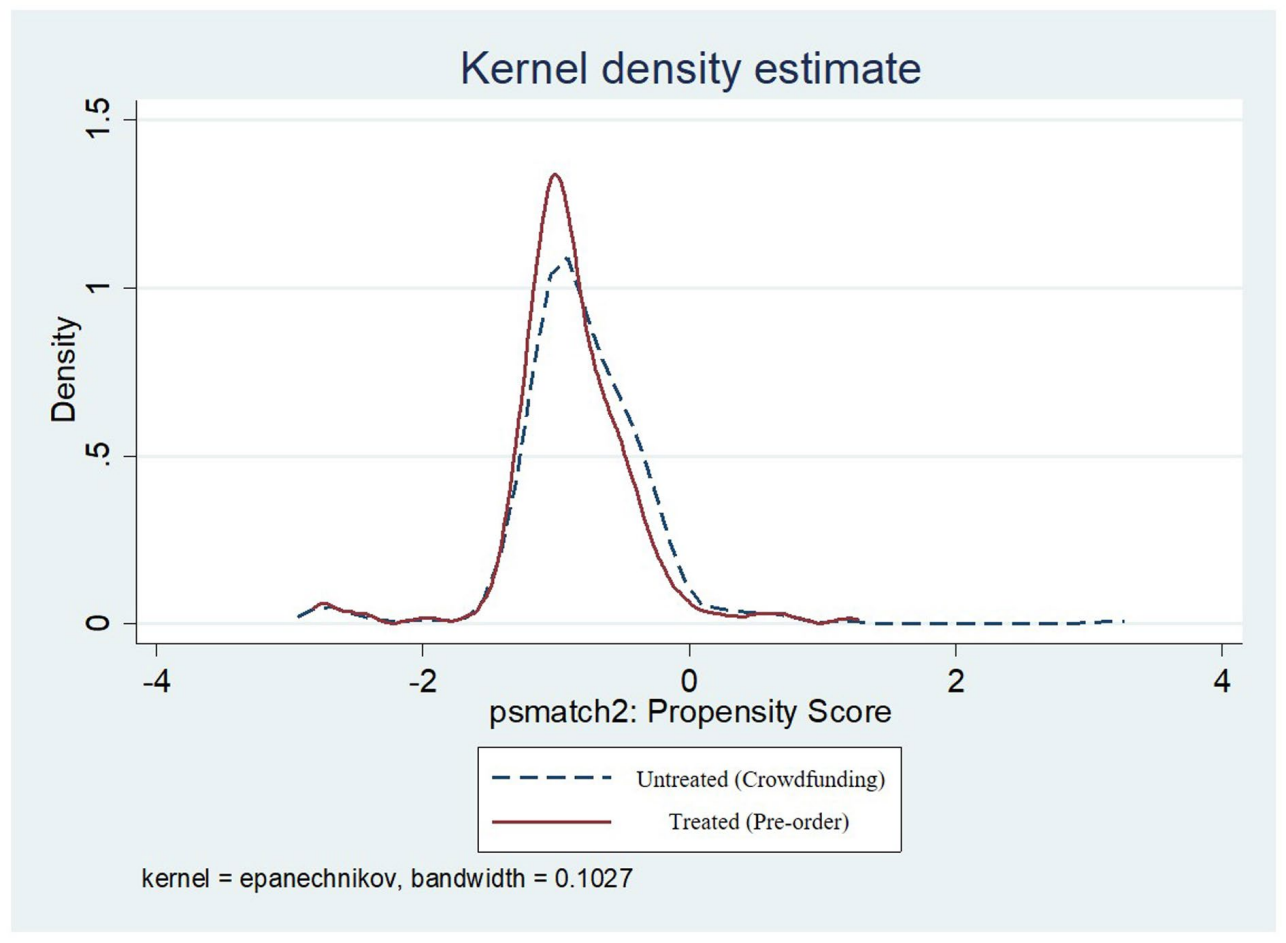
The post matching score density of crowdfunding projects and pre-order items.

In the common support test, kernel density estimation of the project data after matching was obtained by using the Stata graphing method. Figure 3 shows the kernel density curves of the two groups of projects after matching in the crowdfunding stage and pre-order stage. It is evident that after matching the two groups of data, the overlap range of the matching kernel density was large, indicating that the common support area of the two groups of data was large and there was sufficient data for matching. In addition, only 65 observations of the control group were not within the common support, and the remaining 1,735 observations were within the common support. Therefore, the two types of crowdsourcing passed the common support test.

#### Step4: Assessing the matching quality

The results of the PSM estimates of the four crowdfunding performance indicators are provided in Table 5. The success rate, the funding ratio, and the number of backers were significantly higher for the pre-order item compared with the crowdfunding project, and the average investment also increased but was not statistically significant. In particular, the success rate of the pre-order item increased by about 30.43% relative to the crowdfunding project, the funding ratio increased by about 8.16, and the number of backers increased by approximately 1,103. Therefore, except for average pledge, pre-order items perform better than crowdfunding projects in the same platforms in terms of the success rate, the funding ratio, and the number of backers, mostly supporting the proposed hypothesis.

**Table 5:** The estimated results of the PSM for crowdfunding projects and pre-order items.

Performance variable	Sample	Treated	Controls	Difference	S.E.	*T*-test
Success	ATT	0.8141	0.5098	**0.3043** ^***^	0.0338	9.00
RA	ATT	10.9620	2.7985	**8.1635** ^***^	1.3995	5.83
BA	ATT	1556.9919	453.3716	**1103.6204** ^***^	193.7079	5.70
PL	ATT	298.4163	207.0236	91.3927	62.5688	1.46

* *p* ≤ 0.05;

** *p* ≤ 0.01;

*** *p* ≤ 0.001.

ATT refers to the average treatment effect on the treated. ATT is estimated using the bootstrapping method (*N* = 500). The value of bold means significant.

### Robustness check

To ensure the robustness of the results, the K-nearest neighbor matching method (1:2, 1:3, and 1:4 matching), kernel matching method, and local linear regression matching method were also chosen to estimate the average treatment effect of project performance in this study, and the specific test results are given in Table 6. The results show that the success rate, the funding ratio, and the number of backers of the pre-order items were significantly larger than those of the crowdfunding projects under the effect of multiple matching methods, while the average investment was not significantly different. Consequently, the estimation results of this paper are robust.

**Table 6:** PSM results for crowdfunding projects and pre-order items (other methods).

Matching estimator		Performance variable	Sample	Treated	Controls	Difference	S.E.	*T*-test
k-nearest neighbors	1:2	Success	ATT	0.81414	0.5393	**0.2748** ^***^	0.0299	9.19
		RA	ATT	10.9620	2.8952	**8.0668** ^***^	1.3850	5.82
		BA	ATT	1556.9919	460.7831	**1096.2088** ^***^	186.1029	5.89
		PL	ATT	298.4163	208.7061	89.7101	59.7883	1.50
	1:3	Success	ATT	0.8141	0.5303	**0.2839** ^***^	0.0283	10.02
		RA	ATT	10.9620	2.8574	**8.1045** ^***^	1.3794	5.88
		BA	ATT	1556.9919	451.3669	**1105.6250** ^***^	183.0699	6.04
		PL	ATT	298.4163	195.0317	103.3846	58.4380	1.77
	1:4	Success	ATT	0.8141	0.5330	**0.2811** ^***^	0.0273	10.31
		RA	ATT	10.9619	3.3383	**7.6237** ^***^	1.4071	5.42
		BA	ATT	1556.9919	574.2852	**982.7067** ^***^	192.4420	5.11
		PL	ATT	298.4163	198.3956	100.0207	57.7896	1.73
Kernel		Success	Success	0.8138	0.5330	**0.2807** ^***^	0.0237	11.84
		RA	RA	10.9782	3.4791	**7.4992** ^***^	1.3873	5.41
		BA	BA	1559.3725	503.5691	**1055.8034** ^***^	182.9079	5.77
		PL	PL	298.7100	201.1611	97.5489	56.3809	1.73
Local linear regression		Success	Success	0.8141	0.5349	**0.2793** ^***^	0.0338	8.26
		RA	RA	10.9620	3.4668	**7.4952** ^***^	1.3995	5.36
		BA	BA	1556.9919	498.4458	**1058.5461** ^***^	193.7079	5.46
		PL	PL	298.4163	202.7560	95.6604	62.5688	1.53

* *p* ≤ 0.05;

** *p* ≤ 0.01;

*** *p* ≤ 0.001.

ATT refers to the average treatment effect on the treated. ATT is estimated using the bootstrapping method (*N* = 500).

The value of bold means significant.

## Discussion

According to the results provided above, the study found that the fundraising performance of pre-order items was better than that of crowdfunding projects in several respects. First, pre-order items are more likely to be fundraising successes. The descriptive statistics reveal that the average fundraising target set for pre-order items was smaller than that of crowdfunding projects, indicating that, in general, pre-order items require less capital than crowdfunding projects, and their creators do not mainly aim to raise funds but rather to understand market demand, advertise and market, target customers, and other purposes. Such projects are less risky for backers and more likely to raise funds successfully.

Second, a greater proportion of pre-order items are funded. For backers, their main purpose for choosing a pre-order item is to obtain the product described by the project. Therefore, whether the project has been successfully funded currently has little impact on them, which means backers are not affected by the diffusion of responsibility effect and will be influenced by the herding effect for projects with good performance and will continue to make additional investments. Eventually, the fundraising ratio of pre-order items will be larger than that of crowdfunding projects.

Third, the number of backers for pre-order items is much larger. Crowdfunding is funded by the general public who do not have professional knowledge and relevant experience, and most of these groups dislike risks. Pre-order items are more mature than crowdfunding projects and are more secure for backers, so more people choose to support pre-order items.

Fourth, the average investments in pre-order items and crowdfunding projects are comparable and do not show significant differences. This paper argues that the average investment in crowdfunding projects should theoretically be larger than that of pre-order items, owing to the special nature of crowdfunding and the reasons for backers’ willingness to contribute, including helping others and achieving self-worth. The reason the average investment is comparable when backers face crowdfunding projects and pre-order items on the same crowdfunding platform is that the riskiness of crowdfunding projects can negatively affect the average investment to some extent.

### Theoretical implications

This paper makes two main contributions to the crowdfunding literature. On the one hand, our study extends the literature that examines potential determinants of crowdfunding performance. Previous studies have identified many influential factors of crowdfunding performance, such as creator information (e.g., social network, past experiences, and team member) (Zvilichovsky et al., 2015; Butticè et al., 2017; Skirnevskiy et al., 2017; Kim and Viswanathan, 2019; Wang et al., 2021; Liu et al., 2022) and projects description (e.g., duration, goal, video, picture, spelling errors, and linguistic style) (Gerber and Hui, 2013; Courtney et al., 2017; Parhankangas and Renko, 2017; Yang et al., 2020; Kim et al., 2022). Our study extends this stream of research by investigating effects of backer’s preference on crowdfunding performance. In particular, we used the product stage badge to identify pre-order items or crowdfunding projects, and empirically investigate the performance difference between pre-order items or crowdfunding projects. Our findings demonstrate that pre-order items perform better than crowdfunding projects in the same platform, suggesting that backers are risk-averse in the context of crowdfunding.

First, it extends the determinants of crowdfunding performance from project and creator characteristics to backer preferences by investigating the performance differences between pre-order items and crowdfunding projects. Second, the similarities and differences between crowdfunding projects and pre-order items are systematically compared, and investigate the preferences of backers for crowdfunding and pre-order stages when choosing to invest. Third, this research contributes to the effectiveness of the governance mechanism of crowdfunding platforms by empirically examining the effects of the product stage badge on fundraising outcomes. These theoretical contributions are grounded in the empirical analysis of a special dataset that has not been traced in the crowdfunding literature.

On the other hand, our study contributes to the effectiveness of the governance mechanism of crowdfunding platforms. The previous literature on platform governance mechanisms focuses on how to optimize the settings of crowdfunding projects’ webpage content, webpage structure, and participation rules to improve the experience of participants so that the platform can develop stably in the long run, and previous literature has conducted much exploration into related aspects from this perspective (Xiao et al., 2014; Burtch et al., 2015; Yang et al., 2020; Kim et al., 2022; Lin et al., 2022; Liu et al., 2022). However, there is no literature that clearly indicates the informational role of the project stage badge on crowdfunding performance. In this study, we treat the project stage badge as an important information for potential backers to judge the risk of the campaigns. Our PSM results indicate that the project stage badge plays an important informational role in determining crowdfunding performance.

### Practical implications

For crowdfunding platforms, crowdfunding creators, and backers, it is important to examine the similarities and differences between crowdfunding projects and pre-order items, and their implications. First, for the platform, the rules, technical features, cultural norms, and overall industry norms that it establishes will shape the behavior of both creators and backers, ultimately determining whether the crowdfunding market works effectively or succumbs to market failure. Crowdfunding platforms know whether backers prefer crowdfunding or pre-order, which helps them improve the rules and web structure design to attract more crowdfunding participants. Second, for crowdfunding creators, a better understanding of crowdfunding backers’ preferences will help them improve their crowdfunding work and increase the probability of success. Finally, for crowdfunding backers, understanding the similarities and differences between crowdfunding projects and pre-order items deepens their understanding of crowdfunding, and they can thus choose crowdfunding according to their preferences, thereby reducing the problems of “herding behavior” and “adverse selection” caused by information asymmetry.

### Limitations and future research directions

Our study has some limitations, which provide opportunities for further research. First, owing to the specificity of this paper, only the Indiegogo crowdfunding platform was selected for analysis, which is not universal. In fact, other crowdfunding platforms do not have specific project stage markers, but according to the descriptions on the project details page, potential investors can summarize the current project’s development stage by themselves and then make their own judgments. In future research, an examination of backers on other crowdfunding platforms would be useful to render the research results more reliable.

Second, the variety of crowdfunding projects on the Indiegogo platform is diverse, but only the physical projects in the Tech and Innovation category incorporate project stage badges, so the amount of data available for the study was small. The time span of the collected data could be increased in the future, thereby increasing the amount of data available. Moreover, the formation of panel data could be considered for further analysis.

Third, there are different categories of Tech and Innovation projects, such as Audio, Camera Gear, Energy and Green Tech, and so on. This paper does not break down whether the different categories of projects at different stages will have different effects on the behavior of backers. In fact, backers can also be divided into different groups according to the type of projects they invest in, since funding motivation includes the personal interests of investors in the crowdfunding market and groups interested in the same thing may have different investment behaviors.

## Conclusion

To investigate whether backers prefer crowdfunding projects or pre-order items in the crowdfunding market, this study took the Indiegogo platform as the research object, collected and screened 1,800 projects with the project stage badge, and classified them into crowdfunding projects and pre-order items according to their development stage, and applied PSM for data analysis. The results demonstrate significant differences exist in funding choices when backers are faced with projects at different stages of development, and that pre-order items outperform crowdfunding projects on the same crowdfunding platform, as evidenced by higher success rates, increased funding ratios, and more backers. While little difference was found in the average funding amount. Our study is among the first to investigate effects of backer’s preference on crowdfunding performance. However, preference is a broad concept including many different types, e.g., regional preference, gender preference, language preference, and innovation preference. In this study, we only focus on risk preference and selected the project stage badge to measure risk preference, future studies can examine other kinds of backer’s preferences and provides a holistic perspective to understanding backer’s preferences in the context of crowdfunding.

## Data availability statement

The original contributions presented in the study are included in the article/supplementary material, further inquiries can be directed to the corresponding authors.

## Author contributions

This idea was given by YZ and NW. YZ wrote the complete paper. JC analyzed the data. While JC and NW read and approved the final version. All authors contributed to the article and approved the submitted version.

## Funding

This research was supported by the National Science Foundation of China (grant numbers: 72272066 and 71971101) and the Key Project of Philosophy and Social Science Research in Colleges and University of Jiangsu Province (grant number: 2019SJZDA032).

## Conflict of interest

The authors declare that the research was conducted in the absence of any commercial or financial relationships that could be construed as a potential conflict of interest.

## Publisher’s note

All claims expressed in this article are solely those of the authors and do not necessarily represent those of their affiliated organizations, or those of the publisher, the editors and the reviewers. Any product that may be evaluated in this article, or claim that may be made by its manufacturer, is not guaranteed or endorsed by the publisher.
